# Management of a broken modular femoral stem following total hip arthroplasty in a patient with sickle cell disease using an endofemoral trephine reamer: A case report

**DOI:** 10.1016/j.ijscr.2021.02.029

**Published:** 2021-02-10

**Authors:** Mohammad A. Arafah, Abdulrahman H. Almalki, Orfan M. Arafah

**Affiliations:** a Department of Orthopedic Surgery, King Saud University Medical City, Riyadh, Saudi Arabia; b College of Medicine, King Saud University Medical City, Riyadh, Saudi Arabia; c Department of Orthopedic Surgery, King Saud University Medical City, Riyadh, Saudi Arabia

**Keywords:** Arthroplasty, Osteonecrosis, Oosening, Trephine reamer, Sickle celldisease

## Abstract

**Introduction and importance:**

Breakage of the femoral stem with intact bone is a rare complication that has only been reported once. Sickle cell disease (SCD) patients are more prone to variable complications due to the nature of their disease. We discuss how to safely remove a fixed broken stem using an intramedullary trephine reamer to achieve optimal outcome in a SCD patient. It is important to keep an intact femoral cortex during arthroplasty to achieve stable prostheses.

**Case presentation:**

We report a 35 years old SCD male, who complains of left hip pain and decreased activity 18-months following total hip arthroplasty with no history of trauma nor infection. Lab work and radiography showed signs of aseptic loosening and breakage of the femoral stem with no signs of cortical fractures. A trephine reamer was used to extract the stem during revision arthroplasty. Two years post-operative follow up showed improved Harris hip score (HHS) and apparent clinical improvement in function and pain.

**Discussion:**

Multiple femoral stem extraction techniques have been reported in the literature. However, these approaches have variable disadvantages. Using the trephine reamer intramedullary helped to extract the distal femoral stem fragment and preserve the integrity of the femoral cortex. However, caution should be taken to avoid iatrogenic injuries.

**Conclusion:**

Using the trephine reamer to extract a broken femoral stem is a safe technique that preserves the cortical integrity. Complications of the trephine reamer include heat necrosis and iatrogenic fractures. Intra-operative measures can be taken to limit those complications.

## Highlights

• Using a trephine reamer to extract a broken femoral stem is a safe technique that preserves the femoral cortical integrity postoperatively in comparison with other techniques.
• Complications of a trephine reamer include heat necrosis and iatrogenic fractures.
• Intra-operative measures can be taken to limit possible complications of trephine reamer such as using new sharp reamers with the appropriate size, regularly irrigating the intramedullary canal while reaming to avoid heat necrosis, and involving a C-arm to avoid any cut-through in the cortex.
• SCD patients who require arthroplasty at young age are prone to a higher risk of aseptic loosening due to the increased physical activity and functional demands which increases the stress on the implant-bone interface hindering implant-bone integration

## 1 Introduction and importance

A nontraumatic fracture of the modular femoral stem in total hip arthroplasty is rare, especially given that the designs of the prostheses have advanced greatly recently, with a prevalence rate of roughly 0.27%[1,2]. However, the increased demand on joint replacements and the catastrophic effects of such a complex complication make finding safe and less invasive surgical solutions with maximum bone-stock perseverance of high importance as they have a direct impact on the clinical and radiological outcomes [3].

Extracting a fractured component poses a challenge to surgeons especially when there is a loose proximal and a well-fixed distal femoral stem [4]. Multiple solutions were described including femoral cortical window technique, extended femoral osteotomy, and retrograde nail impaction through the knee joint. However, these techniques are either radical, provide limited exposure to the distal fragment, or lead to the loss of distal intra-medullar support [1,2]. In addition, specific instruments are required to achieve a safe extraction of the well-fixed distal stem, including flexible osteotomes, Gigli saws, high-speed burrs, and trephine reamers [3].

The usage of a trephine reamer for the extraction of a broken implant was first described by Collis in 1984 [5] and subsequent studies have shown that the use of a trephine reamer can aid in the extraction of the distal femoral stem safely [3]. However, careful measurements should be made to avoid complications. In the present case, we adopted a trephine endofemoral approach to extract a well-fixed broken distal femoral stem from a sickle cell disease (SCD) patient who had no cortical fracture without violating the cortex.

This paper has been reported in line with the 2020 SCARE criteria [6], and has been registered in the Research Registry.

## 2 Case presentation

**Fig. 1:**
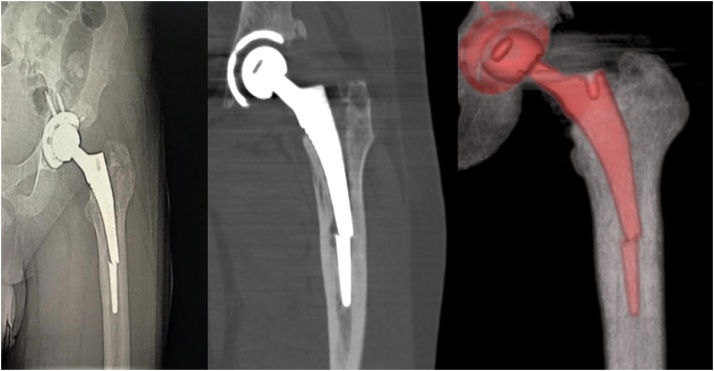
X-rays and CT scans of the hip 18 months after primary total hip arthroplasty.

A 35-year-old Saudi SCD male on hydroxyurea, with no other relevant medial or family history, presented to our university hospital outpatient clinic, complaining of left hip pain 18 months following cementless left total hip arthroplasty using the DePuy Corail Hip System. The patient presented complaining of left-sided thigh pain for one month that had started after performing a sudden motion of the hip, but had no traumatic event or history of infection. Upon examination, the patient displayed no signs of local infection, no tenderness, normal power, and intact distal neurovascular function. Moreover, his left hip range of motion was limited in extreme flexion and internal rotation due to pain. The Harris Hip Score (HHS), which is a 100-point questionnaire used to objectively assess the hip joint function according to four main domains (pain, function, deformity, and range of motion) [13], was 33 points. X-rays and computed tomography showed broken distal femoral stem with no apparent cortical fracture, the proximal part of the femoral stem was loose while the distal fragment remained fixed inside the intramedullary canal (Fig. 1). Lab workup and inflammatory markers were taken and showed no signs of infection. The patient was diagnosed with aseptic loosening of the left femoral stem. He was subsequently scheduled for left-sided femoral stem revision.

### 2.1 Operative technique

**Fig. 2:**
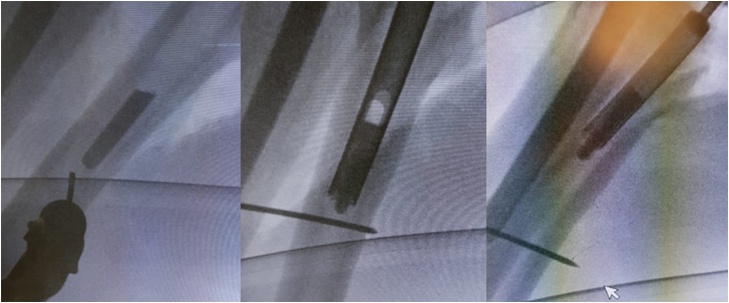
Fluoroscopy images showing a Kirschner wire placed distal to the distal femoral stem fragment to avoid further migration distally, and a trephine reamer was used to extract the distal fragment.

**Fig. 3:**
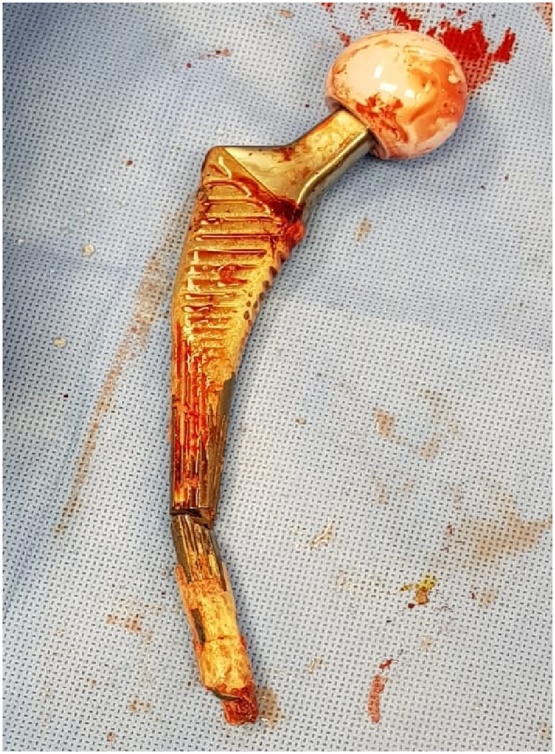
Gross photo of the implant used showing the distal femoral stem fracture and the significant bone osteosynthesis around the distal femoral stem in comparison with the proximal part of the implant.

Multidisciplinary team was involved to manage the patient’s SCD condition and optimize the pre- and postoperative setup. It is important to maintain proper oxygenation and hydration, to prevent hypothermia, and to control the HgbS concentration. The patient underwent left-sided revision arthroplasty using a posterior approach to the hip along the previous surgical incision which was done by a senior orthopedic arthroplasty consultant. Soft tissue biopsy and fluid swabs were taken from the hip joint for frozen section, culture and sensitivity intraoperatively, and were all negative. The joint was then dislocated to address the femoral implant. The liner was intact and the proximal part of the femoral stem and head were removed easily. Afterward, we placed a Kirschner wire distal to the tip of the femoral stem to prevent the stem from migrating distally. Subsequently, a trephine reamer was adopted under guidance of the C-arm to disengage the distal femoral stem, whose size was chosen based on the diameter of the extracted proximal end. While using the trephine reamer, generous irrigation with saline was performed to avoid overheating and osteonecrosis of the bone. Finally, the distal fragment was caught inside the trephine reamer and was removed (Fig. 2). A 16 Wagner SL stem (Zimmer Biomet, Warsaw, IN, USA) was used during the revision procedure. Reduction and stability were confirmed, and since there was no cortical fracture, a prophylactic cerclage wire was not needed prior to stem insertion. A gross image of the extracted implant can be seen in (Fig. 3).

**Fig. 4:**
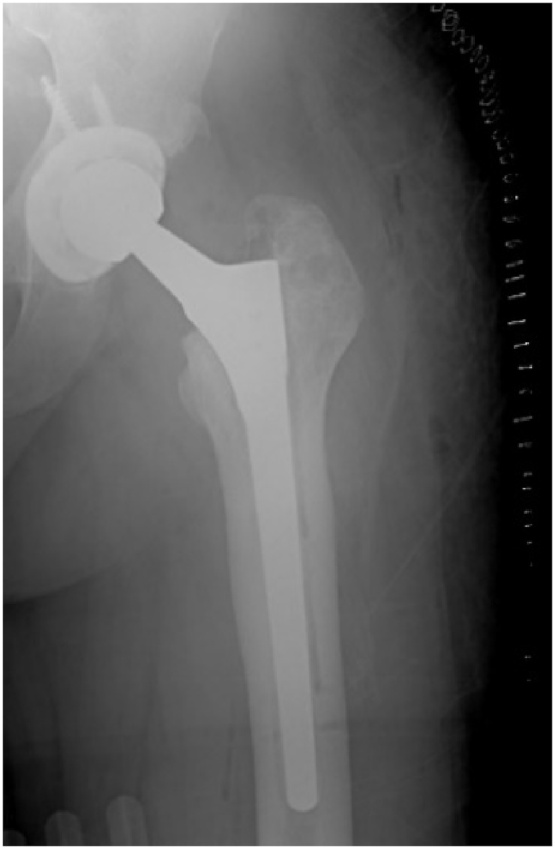
Immediate postoperative X-ray of the left hip arthroplasty performed using the Wagner SL (Zimmer Biomet, Warsaw, IN, USA) implant.

The operation went well with no intraoperative complications. Immediate post-operative X-ray is seen in Fig. 4. Five days after his procedure the patient was discharged and was meeting his physical therapy goals. The patient was instructed to fully weight bear and to avoid crossing his legs or flexing his hip or bending past 90 degrees in the early phase of his rehabilitation to prevent post surgical dislocations.

### 2.2 Postoperative follow-up

**Fig. 5:**
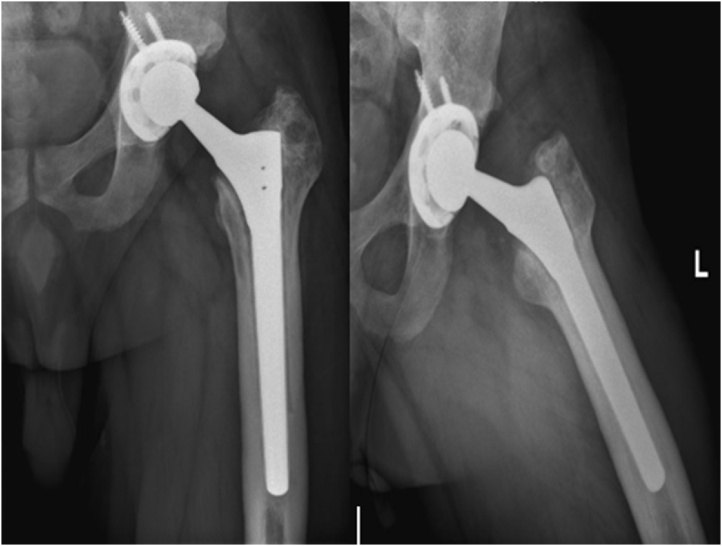
Latest anteroposterior and lateral X-rays of the left hip.

The patient was followed up with regularly without any complications, he had full range of motion and no signs of pain or deformity. His postoperative HHS was 76 points. The patient was able to go back to his normal day activities. However, it is important to mention that the patient required another revision procedure due to aseptic loosening at the distal tip of the implant diagnosed by bone scan. The patient was seen 2 years after his primary revision procedure at our university hospital’s outpatient clinic and was doing well, fully weight-bearing, and has full range of motion and no pain. X-rays showed well-fixed implants with no signs of loosening (Fig. 5). The patient returned to practicing his usual lifestyle with no apparent limitations in his activity, and expressed his happiness to regain full function and return to normal activity in a timely manner.

## 3 Discussion

Failure of the femoral stem is a complication that is rarely described in the literature especially in an atraumatic setting with no signs of femoral bone fracture. To our knowledge, only one case published by Conrad et al. in 2015 reported a similar presentation [8]. Removing the fixed distal femoral stem from within the medullary canal is a challenge during revision procedures. Multiple techniques have been reported in the literature including the utilization of an extended trochanteric osteotomy [9], performing cortical windows [10], or the removal of cement using osteotomes or reamers [11]. However, these approaches have variable disadvantages including violating the normal bone cortex.

Using the trephine reamer intramedullary with the aid of the C-arm helped to extract the distal femoral stem fragment and preserve the integrity of the femoral cortex, minimizing the risks of developing iatrogenic fractures such as those associated with performing cortical windows or using osteotomes. The insertion of a distal Kirschner wire was used to preventing migration of the implant distally. On the other hand, it has been reported that trephine reamers have their own disadvantages in the form of heat necrosis caused by drilling [8] and the breakage of the reamer heads or resultant cortical perforations [12].

To limit such complications, it is essential to confirm the availability of new sharp reamers with the appropriate size needed based on the distal end of the proximal fragment, regularly irrigate the intramedullary canal while reaming to avoid heat necrosis, and involve a C-arm to avoid any cut-through in the cortex. Furthermore, Kancherla et al. recommended using a tapered stem in the revision surgery, bypassing the distal extent of the trephine by a minimum of 4 cm after a trephine has been deployed [3].

Physical examination findings and function were improved significantly post-operatively and his HHS improved from 33 to 76 points [13]. Though the patient required a second revision due to implant aseptic loosening, we believe that this complication occurred as a sequalae of his SCD.

SCD is a common condition in Saudi Arabia. SCD patients from the eastern province have a greater risk of developing avascular necrosis of the femoral head (average of 27%) in comparison with patients from other regions (average of 10%) [14]. These patients are prone to multiple medical morbidities.

S.C. Fassihi et al. conducted a recent systematic review to analyze the outcomes (HHS, duration of hospital stay, post op pain) and complications of total hip arthroplasty in SCD patients in comparison to non-SCD patients. In their review, it was noted that SCD patients who require arthroplasty at young age, just like our patient, are prone to a higher risk of aseptic loosening due to the increased physical activity and functional demands, which increases the stress on the implant-bone, interface hindering implant-bone integration [15]. However, it is always important to rule out any infectious disease first as SCD patients are at higher risk of developing infections due to functional asplenia as part of the disease’s sequelae.

## 4 Conclusion

The use of a trephine reamer for extracting a fractured distal femoral stem is a safe technique that preserves the femoral cortex postoperatively in comparison with other techniques, and yields promising functional outcomes. To limit possible complications, preoperative planning and intraoperative considerations need to be taken.

## Declaration of Competing Interest

None.

## Funding

None.

## Ethical approval

The Institutional Review Board at King Saud University Medical City, Riyadh, Saudi Arabia has exempted the study from an ethical approval.

## Consent

Written informed consent was obtained from the patient for publication of this case report and accompanying images. A copy of the written consent is available for review by the Editor-in-Chief of this journal on request.

## Author contribution

Mohammad Arafah: study design, data collection, literature review, writing and submission, editing reviews.

Abdulrahman AlMalki: data collection, literature review, writing and drafting of manuscript.

Orfan Arafah: study conception, study analysis, critical revision, supervision, reviewing and editing.

## Registration of research studies

researchregistry6538: https://www.researchregistry.com/register-now#home/registrationdetails/601e7ec1e7937e001b701445/

Guarantor

Dr. Orfan M. Arafah MBBS, FRCSC, MHsC, Orthopedic Consultant.

## Provenance and peer review

Not commissioned, externally peer-reviewed.
